# Intercellular mitochondrial transfer in the brain, a new perspective for targeted treatment of central nervous system diseases

**DOI:** 10.1111/cns.14344

**Published:** 2023-07-09

**Authors:** Ziang Geng, Shu Guan, Siqi Wang, Zhongxue Yu, Tiancong Liu, Shaonan Du, Chen Zhu

**Affiliations:** ^1^ Department of Neurosurgery Shengjing Hospital of China Medical University Shenyang China; ^2^ Department of Surgical Oncology and Breast Surgery The First Hospital of China Medical University Shenyang China; ^3^ Department of Radiation Oncology The First Hospital of China Medical University Shenyang China; ^4^ Department of Cardiovascular Ultrasound The First Hospital of China Medical University Shenyang China; ^5^ Department of Otolaryngology Shengjing Hospital of China Medical University Shenyang China; ^6^ Department of Neurosurgery The First Hospital of China Medical University Shenyang China

**Keywords:** central nervous system, mitochondrial transfer, targeted therapy

## Abstract

**Aim:**

Mitochondria is one of the important organelles involved in cell energy metabolism and regulation and also play a key regulatory role in abnormal cell processes such as cell stress, cell damage, and cell canceration. Recent studies have shown that mitochondria can be transferred between cells in different ways and participate in the occurrence and development of many central nervous system diseases. We aim to review the mechanism of mitochondrial transfer in the progress of central nervous system diseases and the possibility of targeted therapy.

**Methods:**

The PubMed databank, the China National Knowledge Infrastructure databank, and Wanfang Data were searched to identify the experiments of intracellular mitochondrial transferrin central nervous system. The focus is on the donors, receptors, transfer pathways, and targeted drugs of mitochondrial transfer.

**Results:**

In the central nervous system, neurons, glial cells, immune cells, and tumor cells can transfer mitochondria to each other. Meanwhile, there are many types of mitochondrial transfer, including tunneling nanotubes, extracellular vesicles, receptor cell endocytosis, gap junction channels, and intercellular contact. A variety of stress signals, such as the release of damaged mitochondria, mitochondrial DNA, or other mitochondrial products and the elevation of reactive oxygen species, can trigger the transfer of mitochondria from donor cells to recipient cells. Concurrently, a variety of molecular pathways and related inhibitors can affect mitochondrial intercellular transfer.

**Conclusion:**

This study reviews the phenomenon of intercellular mitochondrial transfer in the central nervous system and summarizes the corresponding transfer pathways. Finally, we propose targeted pathways and treatment methods that may be used to regulate mitochondrial transfer for the treatment of related diseases.

## Graphical abstract

Different cells undergo mitochondrial transfer through different methods, including vesicle‐mediated mitochondrial transfer, PPARγ‐mediated mitochondrial transfer, gap junction‐mediated mitochondrial transfer, and TNT‐mediated mitochondrial transfer. Meanwhile, different transfer methods involve different molecular pathways.

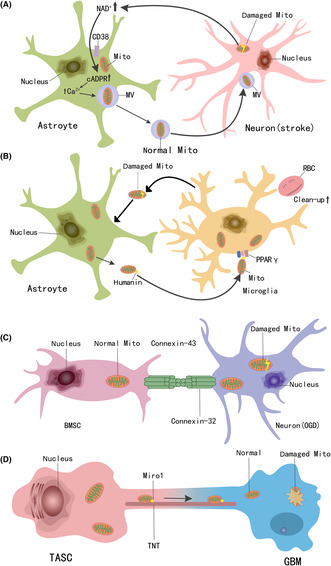

## 1 INTRODUCTION

Mitochondria are the most complex organelles found in eukaryotic cells and are different from those seen in prokaryotes. Eukaryotic mitochondria are composed of a double outer membrane and their genome (mtDNA), which contains circular DNA, can replicate independently of the host genome. In animal cells, the mtDNA is small (10–39 kb),^1^ where it encodes for 2 rRNAs (12S and 16S), 22 tRNAs, and 13 polypeptides, with each polypeptide containing approximately 50 amino acid residues. Although mtDNA can synthesize proteins, this ability is very limited where most of the proteins encoded by mtDNA are related to oxidative phosphorylation (OXPHOS). However, proteins that constitute other parts of the mitochondria are encoded by nuclear DNA and synthesized by cytoplasmic ribosomes before being transported to their respective functional sites within the mitochondria. Therefore, mitochondria remain reliant on nuclear DNA for many of its structural and functional proteins and are semi‐autonomous organelles.^2^ Mitochondria are generally considered as the energy source of eukaryotic cells including the production of adenosine triphosphate (ATP). As well as this important function, mitochondria also participate in key central metabolic pathways and are fully integrated into the intracellular signaling network that regulates a variety of cell functions, playing an important role in maintaining cell homeostasis.

Mitochondria also play an important role in many processes, including mtDNA repair,^3^ reactive oxygen species (ROS) production,^4^ mitochondrial biogenesis, dynamics,^5^ fusion,^6^ and mitochondrial proteomic regulation; defects in these processes may lead to disease states. Mitochondrial dysfunction can trigger an increase in ROS production and the activation of apoptosis pathways, leading to the occurrence and development of many diseases.^7^ In addition, during stress, the regulatory mechanism of cells cannot maintain mitochondrial homeostasis, leading to their dysfunction. The central nervous system is composed of the brain and spinal cord and at the cellular level, it contains neurons, glial cells, and other types of cells. The central nervous system governs the physiological activities of all systems in the body and plays an important role in the response and regulation of the whole system, which cannot be separated from the integrity and activity of mitochondria. In the damaged or diseased brain, mitochondrial dysfunction leads to decreased ATP levels, which impairs ATP‐dependent neuronal firing and neurotransmitter dynamics. At the same time, mitochondrial dysfunction can also trigger an increase in ROS production and the activation of related apoptosis pathways.^8^ At present, varied mitochondrial dysfunction has been found in ischemic diseases,^9^ stress injury, schizophrenia, Parkinson's disease,^10^ Alzheimer's disease,^11^ intracranial hemorrhage,^12^ spinal cord injury, inflammatory pain, autoimmune encephalopathy, and intracranial tumors. Therefore, the restoration of normal biological function in damaged mitochondria is one of the research hotspots for the treatment of neurological diseases.

Since the first proposal of intercellular mitochondrial transfer, it has been studied by an increasing number of researchers. Spees et al.^13^ first observed the phenomenon of mitochondrial transfer in 2006, in human epithelial cells with abnormal mitochondrial function (A549 cells) using fluorescently labeled mitochondria from mesenchymal stem cells (MSC) in a co‐culture system. They found that mtDNA from donor MSCs appeared in human epithelial cells and restored their respiratory function. From this, numerous studies have confirmed that mitochondria can indeed be transferred between cells by different mechanisms, and alter the biological activity and physiological state of recipient cells. The transfer of normal mitochondria from donor cells to recipient cells with abnormal mitochondrial functions can increase mitochondrial‐related biosynthesis in recipient cells, and then affect the biological functions of the recipient cells.^14^ Therefore, this manuscript systematically reviews the phenomenon of intercellular mitochondrial transfer in the central nervous system and summarizes the relevant transfer pathways, corresponding signaling pathways, and molecular mechanisms involved. Finally, we propose that this process and its intervention may represent new targets for the development of therapeutic agents.

## 2 CELL TYPES INVOLVING INTERCELLULAR MITOCHONDRIAL TRANSFER WITHIN THE CENTRAL NERVOUS SYSTEM

### 2.1 Stem cells to neuronal cells

Stem cells are characterized by the ability of unlimited self‐renewal, resulting in at least one type of highly differentiated progeny cell. Most scholars believe that stem cells are derived from embryos, fetuses, or adults with unlimited self‐renewal, proliferation, and differentiation under certain conditions. They can produce daughter cells with the same phenotype and genotype as the original, and can also produce cells that constitute body tissues where specialized cells in organs can also differentiate into progenitor cells. Stem cells can be divided into embryonic or adult stem cells. Embryonic stem cells refer to cells selected from cell clusters in embryos or primordial germ cells through inhibition culture methods in vitro. Adult stem cells refer to undifferentiated cells existing in a differentiated tissue; these can self‐renew and specifically form cells of the resident tissue. Adult stem cells exist in various tissues and organs of the body and include hematopoietic stem cells, bone marrow mesenchymal stem cells (MSC), and neural stem cells. At present, most of the research related to mitochondrial transfer in the nervous system is based on adult stem cells.

MSCs are stem cells with the potential for self‐replication and multi‐directional differentiation. MSCs can exist in the nervous system. Under specific conditions, MSCs can not only differentiate into mesoderm cells but also horizontally differentiate into glial cells and neurons derived from ectoderm.^15^ When pathological signals appear in the brain, MSCs can migrate to the lesion and perform related functions. MSCs can alleviate neuronal functional damage after cerebral ischemia, secrete a series of factors and differentiate into functional neurons, playing a significant role in repairing damaged neurons and behavioral damage caused by ischemia.^16^ In GBM, mesenchymal stem cells exist in the tumor microenvironment of glioma, and their existence is related to the survival of patients and the invasiveness of GBM.^17^ MSCs can promote the repair of various tissue injuries due to their unique biological characteristics, such as self‐renewal, hematopoietic support, nutritional provision, activation of endogenous stem/progenitor cells, differentiation and transdifferentiation, immune regulation and inflammatory response, anti‐apoptosis, anti‐oxidation, anti‐fibrosis and the promotion of angiogenesis. Among these, mitochondrial transfer is a newly discovered mechanism in MSCs. Studies have shown that when MSCs act as mitochondrial donors and are co‐cultured with other cells, the mitochondria can be transferred from the MSCs to recipient cells in different ways.^18^ Many experiments have found and confirmed that MSCs can connect with recipient cells by forming tunnel nanotubes to promote the transfer of mitochondria to smooth muscle cells.^19^ Similarly, some studies have shown that MSCs can also transport self‐released vesicles which were wrapped with mitochondria to alveolar epithelial cells through gap junction channels, and these vesicles can be absorbed by receptor cells through endocytosis.^20^ MSCs can also directly release active mitochondria under the stimulation of inflammatory factors, which can also be directly absorbed by receptor cells through endocytosis.^21^ These studies have shown that MSC‐derived mitochondria can change the bioactive state of recipient cells and have found that multipotential mesenchymal stem cells (MMSC) can transfer mitochondria to nerve cells or glial cells. These MMSCs can directly transfer mitochondria to normal neurons,^22^ and astrocytes. These studies transfected MMSCs with a lentiviral construct encoding a red fluorescent protein (mitodsred) fused with a mitochondrial localization signal. After 2 days of co‐culture with the two cells, red fluorescence‐labeled mitochondria were seen in astrocytes, which were confirmed to be derived from MMSC. At the same time, researchers also found that when oxygen‐glucose deprivation (OGD) in astrocytes or neuron‐like PC12 cells was used to simulate ischemic injury in the nervous system related to the elevation of ROS, the MMSCs increase the number of mitochondria transferred to the above two cell types. The result of this transfer was to restore the respiratory function of recipient cells and stimulate their proliferation to compensate for mitochondrial dysfunction and cell death caused by related injuries.^23^ Human umbilical cord‐derived MSCs can also be used as mitochondrial donor cells. Here, a cell model of Alzheimer's disease was established using okadaic acid (OA) treated SH‐SY5Y neural cells. After the addition of the treated neural cells to the medium containing human umbilical cord‐derived MSCs for co‐culture, it was found that the MSCs could release extracellular vesicles and transfer mitochondria into neural cells, as determined by flow cytometry. After the transfer of mitochondria, the p181 tau level in the SH‐SY5Y cells was significantly reduced, and the mitochondrial oxidative stress was also alleviated. The results show that human umbilical cord‐derived MSCs can inhibit apoptosis and improve mitochondrial function by transporting mitochondria in the form of extracellular vesicles into OA‐treated SH‐SY5Y neural cells.^24^

### 2.2 Neuronal glial cells to neuronal cells

**FIGURE 1:**
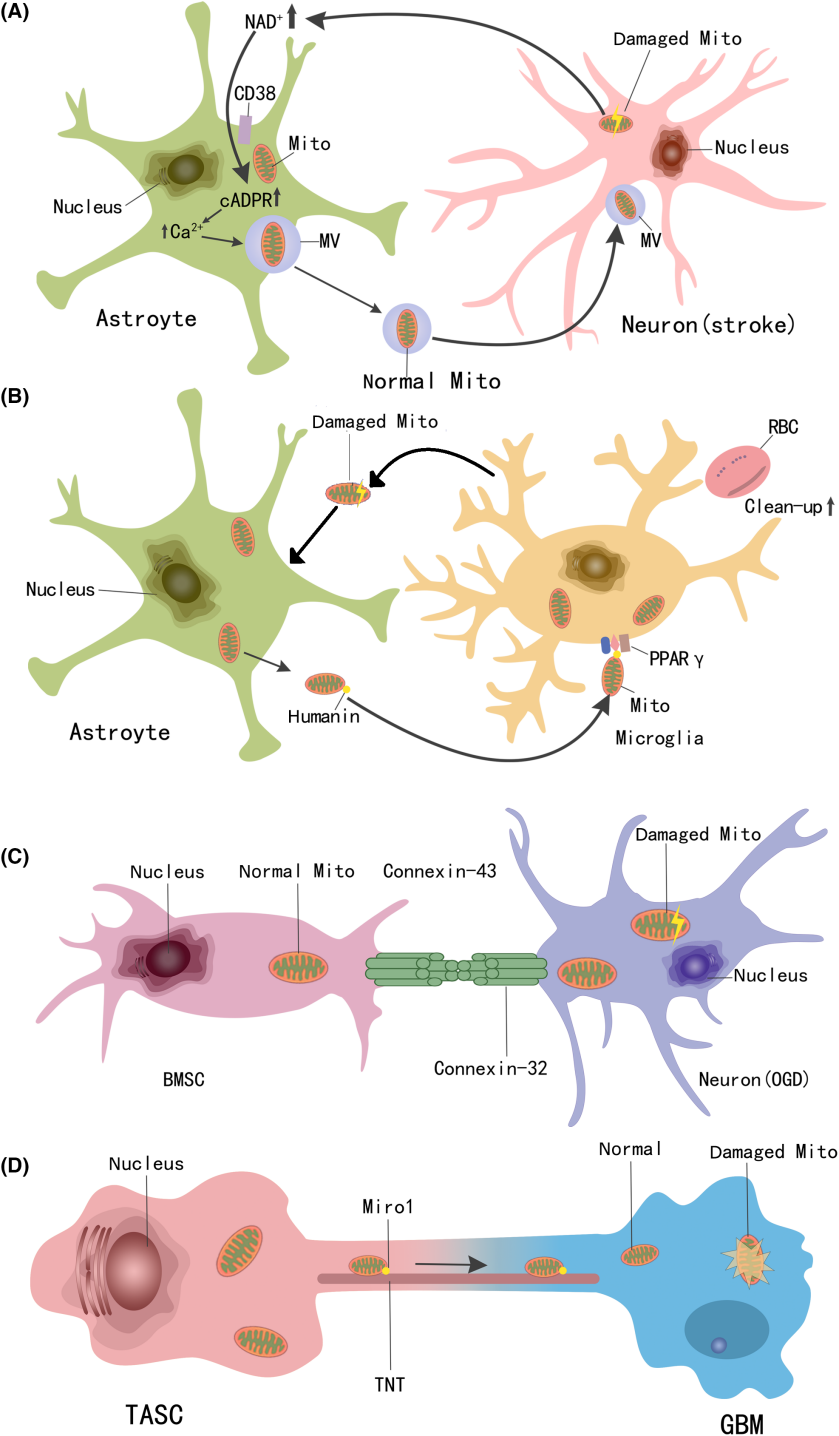
Multiple modes and mechanisms of mitochondrial transfer in different cells. (A) Microvesicles‐mediated mitochondrial transfer, (B) humanin‐PPARγ‐mediated mitochondrial transfer, (C) gap junction‐mediated mitochondrial transfer, (D) tunneling nanotubes‐mediated mitochondrial transfer.

The central nervous system, in addition to nerve cells, which account for the largest proportion of cells, also contains glial cells. There are four types of glial cells, astrocytes, oligodendrocytes, microglia, and ependymal cells. They provide logistical support for the activities of neurons. Astrocytes play an important role in the central nervous system, and they participate in the regulation of important activities such as development, neurotransmission, and neuronal metabolism.^25^ Astrocytes also play an important role in protecting neurons from oxidative stress and exogenous toxicity, and the mechanism that produces these protective effects is likely to be an influx of healthy mitochondria.^26^ Researchers speculate that astrocytes can release healthy functional mitochondria, protect damaged neurons from apoptosis by transferring them to neural cells damaged by ischemic stress, and thus support the survival of neurons after ischemic stroke (Figure 1A). Researchers found that astrocytes can deliver 300–1100 nm mitochondrial particles, and mitochondrial fluorescence labeling showed that these mitochondria retained normal activity.^27^ In a cell model of simulated ischemic injury, cortical neurons from rats were deprived of oxygen and sugars, causing a decrease in intracellular ATP content and neuronal viability. When an astrocyte culture medium containing extracellular mitochondrial particles was added to neurons with simulated ischemic injury, it was found that the ATP content in the neurons increased, and fluorescent labeling confirmed the existence of astrocyte‐derived mitochondria. This experimental result confirmed a role for astrocytes in the protection of neuronal cells against ischemia by mitochondrial transfer, thus avoiding apoptosis.^27^

In a different study, researchers also found experimental evidence of astrocytes transferring mitochondria to nerve cells. Researchers used cisplatin to culture primary cortical neurons to simulate an in vitro model of neuronal damage caused by external chemical privacy. After treatment with cisplatin, the survival rate of neurons decreased significantly, this may be due to a decrease in the depolarization of the mitochondrial membrane potential, which led to abnormal calcium dynamics and increased calcium ion levels in neurons at rest. After the co‐culture of astrocytes and neurons which were treated by cisplatin, the survival rate of the neurons was significantly improved, the mitochondrial membrane potential was increased, and the respiratory efficiency was significantly improved. Mitochondria in astrocytes were labeled using a mitochondrial localization sequence coupled with mCherry. Under confocal microscopy, astrocyte‐derived mitochondria were found in neuronal cells. Cisplatin‐induced an approximately three‐fold increase in the percentage of neurons receiving astrocyte mitochondria when compared to healthy neurons. This mitochondrial transfer can change the calcium concentration state in damaged neurons. The transfer of mitochondria from astrocytes to neurons plays an important role in restoring the KCL‐induced increase in calcium ions in cisplatin‐injured neurons and normalizing neuronal calcium dynamics. This transfer is mediated by Miro1, and the relevant pathways will be described in detail later.^28^

### 2.3 Immune cells to neuronal cells

Immune cells are an important part of the immune system and participate in various immune activities of the human body. Macrophages are a type of immune cell with great plasticity. Their main function is to induce tissue healing and have a strong anti‐inflammatory function. Macrophages can be divided into inactivated M0‐type macrophages and activated M1 and M2‐type macrophages. Here the different subtypes have different immune roles and interestingly, the central nervous system has its own macrophages, termed microglia. When compared with microglia, peripheral nerve tissue gives resident macrophages their unique characteristics.^29^ After nerve injury, macrophages may be recruited to the site of injury to exert anti‐inflammatory functions and some studies have shown that M1 macrophages can accumulate in the dorsal root ganglia of the spinal cord, leading to neuropathic pain.^30^ However, macrophages can also alleviate inflammatory pain, and this mechanism may be due to changes in the intracellular environment of the spinal dorsal root ganglion by mitochondrial transfer. These researchers used carrageenan and injected it into the spinal dorsal root ganglia of mice to simulate inflammatory pain and found that macrophages could infiltrate into the spinal dorsal root ganglia. However, the macrophages were not directly recruited to the site of inflammation but gathered away from the inflammatory site to actively control inflammatory pain. During remission of the inflammatory pain in mice, the M2‐like macrophages infiltrated the dorsal root ganglia containing the sensory neuronal bodies, while oxidative phosphorylation in sensory neurons was restored. Pain relief and mitochondrial transfer require the expression of the CD200 receptor (CD200R) on macrophages and the non‐standard CD200R ligand iSec1 on sensory neurons. Macrophages secrete outer vesicles containing mitochondria carrying CD200R, which combined with the spinal dorsal root ganglia expressing iSec1 on the cell membrane to transfer mitochondria into nerve cells. These data indicate a new mechanism for actively alleviating inflammatory pain, based on mitochondrial transfer.^31^

### 2.4 Between neuronal cells or neuron glial cells

From the above description, we can see that a variety of cells can transfer mitochondria into nerve cells, thereby changing their physiological state. There is also the phenomenon of mitochondrial transport between nerve cells or glial cells. Previous studies have shown that after ischemic stroke, nerve cells in the brain will produce a series of complex pathophysiological events, including excitotoxicity, oxidative stress, inflammatory infiltration, and apoptosis of the ischemic neurons.^32^ Oxidative stress caused by ischemic injury will further lead to mitochondrial damage, which then leads to the emergence of new oxidative stress, resulting in a vicious cycle.^33^ In the face of ischemic injury to nerve cells, glial cells can transfer healthy mitochondria into nerve cells, thereby improving the survival rate of the damaged cells, which has been mentioned in the above description. Mitochondria can also be exchanged between nerve cells. N2a neural cells were treated with hydrogen peroxide (H_2_O_2_) to simulate cerebral ischemic events in the nervous system. The neurons treated with H_2_O_2_ showed intracellular mitochondrial damage and mitochondrial DNA deletions. Then there were co‐cultured with normal N2a neural cells. Through confocal microscopy, it was found that the mitochondria could be transferred from normal N2a cells to damaged or missing N2a cells through tunnel nanotubes. Mitochondria derived from healthy cells could then protect N2a cells from H2O2‐induced apoptosis. The experimental results show that the transfer of mitochondria between nerve cells reduces the production of ROS and avoids damage and apoptosis.^34^

Intracerebral hemorrhage (ICH) is a common condition seen in brain injury, which belongs to a subtype of stroke caused by cerebral vascular rupture. Because of its acute onset and uncertain bleed location, it has a high mortality rate and a poor prognosis.^35^ After cerebral hemorrhage, the blood will directly contact the brain parenchyma, and chemical components in the blood can stimulate this brain parenchyma, leading to biochemical damage to nerve cells, including oxidative damage and complex inflammatory reactions.^36^ As the condition progresses, the hematoma is gradually cleared by microglia through phagocytosis. As mentioned, microglia are macrophages within the nervous system, and this phagocytosis may be activated by peroxisome proliferators activating receptor γ (PPARγ). This transcription factor regulates the expression of hematoma clearance‐related proteins, including superoxide dismutase and catalase.^37^ Impaired expression and dysfunction of antioxidant enzymes may aggravate oxidative damage in brain tissues affected by ICH, where the level of the essential mitochondrial antioxidant enzyme manganese superoxide dismutase (Mn‐SOD) is reduced. Therefore, upregulating the level of healthy mitochondria in the area around the hematoma may alleviate cell damage mediated by ICH. Humanin (HN) is one type of ligand for PPARγ and is a polypeptide composed of 24 amino acids, which is transcribed and translated from mitochondrial DNA. HN can be stored in mitochondria as a mitochondrial autocrine, paracrine, or endocrine signal, or secreted to the outer mitochondrial and extracellular spaces.^38^ Therefore, it is speculated that microglia can bind their own PPARγ by internalizing foreign mitochondria (Figure 1B). The level of HN in hematoma is increased, which enhanced clearance and phagocytosis. Researchers first confirmed that astrocytes can release mitochondrial particles through co‐culture and fluorescent labeling studies, and these mitochondrial particles can be internalized by microglia. They also measured the content of HN in microglia, and the results showed that astrocytic mitochondria accumulated in microglia could increase the level of HN in these cells. Moreover, internalized mitochondria and their secreted HN can further promote the expression of PPARγ on microglial cell membranes. To verify whether the above changes can enhance phagocytic function in microglia, researchers used HN to treat microglia alone, and then added red blood cells as phagocytic target cells and the number of red blood cells was recorded. The results showed that HN effectively increased the number of red blood cells phagocytized by microglia. Therefore, all of the above experimental results showed that during ICH, astrocytes can provide mitochondria to microglia, which can then secrete more HN and stimulate microglia to increase membrane PPARγ. HN combines with PPARγ on the cell membrane surface of microglia through autocrine effects and enhances the phagocytosis of red blood cells in the hematoma by microglia, accelerating clearance of ICH, and protecting the nervous system from further damage.^39^

The above discussion mentioned that mitochondria can transfer from astrocyte to microglia, affecting the phagocytic function of microglia, while microglia can also transfer mitochondria to astrocyte, thus promoting or inhibiting the occurrence of neuroinflammation. In neurodegenerative diseases, there is a cascade effect of neuronal necrosis, in which the degenerated or necrotic neurons activate peripheral glial cells, causing more neuronal degeneration or necrosis through certain means, leading to the progression of the disease. In neurodegenerative diseases, a common feature of Alzheimer's disease (AD), amyotrophic lateral sclerosis (ALS), and Huntington's disease (HD) is the accumulation of neurotoxic proteins in neurons, leading to neuronal dysfunction and ultimately neuronal death.^40^ In these diseases, dysfunctional mitochondria released by glial cells in a pro‐inflammatory state can activate glial cells of the same type or other types, causing them to become in a pro‐inflammatory state, further leading to neuronal damage.^41^ The mitochondrial disruption caused by mitochondrial fission induced by dynein‐related protein 1 (Drp1) is a typical cell feature of degenerative diseases. Blocking Drp1‐mediated mitochondrial fission can prevent neuronal degeneration.^42^ In neurodegenerative diseases, fragments of dead neurons are believed to trigger neuroinflammation mediated by glial cells, thereby increasing neuronal death. One study showed that the expression of neurotoxic proteins related to these diseases in microglia could directly trigger the death of naïve neurons, and spread neuronal death by activating naïve astrocytes to the A1 state. The spread of injury is largely mediated by the release of mitochondria from fragmentation and dysfunctional microglia to the neuronal environment. For normal mitochondria, the number of damaged mitochondria released by microglia and the subsequent neuronal damage is determined by mitochondrial breaks mediated by Drp1‐Fis1 in glial cells.^43^ Researchers found that microglia can be directly activated by neurotoxic proteins, and its mechanism depends on the excessive mitosis of mitochondria‐mediated by Drp1‐Fis1. When microglia are activated by neurotoxic protein co‐cultured with the original astrocyte, activated microglia can release dysfunctional mitochondria, including changes in mitochondrial membrane potential and loss of cytochrome c, transport to naïve astrocyte, and activate astrocyte to A1 state. Astrocyte in the A1 state can also release corresponding cytotoxic factors or dysfunctional mitochondria and transmit them to neurons, eventually leading to neuronal death, that is, extracellular dysfunctional mitochondria will spread damage from microglia to astrocyte and then to neurons. This study proved that microglia can release damaged mitochondria, transfer them to astrocyte, activate astrocyte, and affect the function of neurons, thereby affecting the development of neurodegenerative diseases.

### 2.5 Mitochondrial metastasis involving tumor cells of the central nervous system

In 1927, Warburg proposed a metabolic process in tumor cells, whereby changes in energy metabolism caused by cell respiration defects can occur in tumor cells. Specifically, the oxidative phosphorylation process that provides a large amount of energy for tumor cells is transformed into aerobic glycolysis. Warburg observed a strange phenomenon in cancer cells whereby under aerobic conditions, glycolysis, and lactic acid content of tumor cells increased significantly, but oxidative phosphorylation did not. This aerobic glycolysis phenomenon is now known as the called Warburg effect.^44^ In recent years, researchers have pointed out that the reprogramming of energy metabolism is the core marker of cancer cells, and this metabolic change may be caused by altered mitochondria.^45^ Some studies have found that there is a large number of mitochondrial metastases in tumors, which can cause metabolic reprogramming. However, unlike the other diseases mentioned above, it has not been clear whether receiving exogenous mitochondria has a protective or inhibitory effect on tumor cells. In some tumors, mitochondrial internalization may help rescue intracellular energy metabolism and increase the sensitivity of tumor cells to radiotherapy.^46^ However, in malignant tumors of the central nervous system, the situation is the opposite. Glioblastoma multiforme (GBM) is the most common intracranial primary malignant tumor in adults, with a high mortality rate. The current gold standard for the treatment of GBM is surgical resection combined with radiotherapy or chemotherapy.^47^ Strong invasiveness is one of the characteristics of GBM, which also leads to a poor prognosis. Studies have found that the Warburg effect mentioned above may be one of the reasons driving the invasiveness of GBM.^48^

Increasingly researchers have found that the influence of tumor genesis and development is not only dependent on the tumor cells themselves but also on the surrounding cells and their environment, collectively termed the tumor microenvironment. Experts believe that the occurrence and development of tumors depend upon the interaction between tumor cells and the tumor microenvironment.^49^ Based on this theory, researchers have speculated that cells within the tumor microenvironment may provide mitochondria to tumor cells to change the metabolic pathway of the tumor cells and affect their biological activities. In the tumor microenvironment of glioma, tumor‐activated stromal cells (TASC) can generate intercellular communications with glioma cells. TASCs are recruited into the glioma microenvironment through factors released by the tumor cells, thus supporting biological processes such as glioma occurrence and development, and treatment resistance.^50^ The reason for these effects is that TASC can transfer mitochondria to glioma cells (Figure 1D). This was found when researchers co‐cultured TASC with GBM cell lines and found that TASC can reduce ROS levels in GBM cells. The metabolic analysis of GBM cells using hippocampal techniques showed that co‐culture could significantly increase the aerobic glycolysis level of GBM cells, but oxidative phosphorylation did not change significantly. Next, the researchers labeled the mitochondria in TASC with mitotic crimson and observed the intercellular communication between TASC and GBM in the co‐culture system through a time‐lapse microscope. They found that tunnel nanotubes (TNT) can be formed between TASC and GBM cells and that mitochondria can be transferred from TASC to GBM cells via these tunnels. Mitochondria after transfer can increase aerobic glycolysis of GBM cells, promote cell proliferation and enhance radiation resistance.^51^

Mitochondria can not only transfer from other cells to glioma cells but also from glioma cells to other adjacent cells. This phenomenon can induce cells around the tumor tissues to adapt to microenvironmental changes caused by tumorigenesis. Under co‐culture and stress conditions, GBM cells and adjacent astrocytes can still form TNTs. Mitochondria in GBM cells can also be transferred to astrocytes through TNTs and These transferred mitochondria are amplified or fused, and contain genetic variations in their mtDNA, leading to the transition of metabolism in astrocytes into a tumor‐like metabolism, that is, more dependent on glucose and glutamine. At the same time, the transferred mitochondria can also protect peritumoral astrocytes from hypoxia and apoptosis.^52^ Mitochondrial exchange among various cells in tumor tissue promotes the occurrence and development of the tumor and maintains the activity of tumor cells.

## 3 PATHWAYS OF INTERCELLULAR MITOCHONDRIAL TRANSFER IN THE CENTRAL NERVOUS SYSTEM

### 3.1 Tunneling nanotubes

A Tunneling nanotube (TNT) is a linear membranous channel that mediates intercellular information transmission. It is composed of a cell membrane and a cytoskeleton component mainly composed of fibrous actin (F‐actin) and microtubulin.^53^ These were first discovered and reported by Rustom et al. in 2004. Using a co‐culture system of human 293 cells and rat PC12 cells, researchers found that there was a delivery tube between the two cells, and its structure was composed of long and thin filaments.^54^ Subsequently, an increasing number of cell types were found to have TNTs, including rat astrocytes, C6 glioma cells, immune cells, and muscle cells.^55^ It is speculated that TNTs may be a common communication mode among mammalian cells where their length and diameter vary considerably. The length can range from several to tens of micrometers, and the diameter can range from tens of nanometers to several micrometers. At present, there is no standardized classification for TNTs, and some scholars divide them into two types according to diameter, with 0.7 microns as the boundary. TNT connection between cells is characterized by dynamics and heterogeneity, meaning that TNTs are always in a continuous state of flux where their formation and disappearance can be only a few minutes apart.^54^ Their heterogeneity implies that their components may originate from both donor and recipient cells. TNTs are not a purely in vitro phenomenon, which is also observed in vivo. TNTs were also found in the mouse heart between cardiomyocytes and fibroblasts,^56^ some human tumors,^57^ and in dendritic cells of the eye cornea.^58^

In the above description, we have mentioned that astrocytes can act as mitochondrial donor cells into glioma cells (Figure 1D). Astrocytes play an important role in the formation of the blood–brain barrier and synapses, and can also participate in the regulation of inflammatory reactions in the nervous system.^59^ Above, we mentioned that astrocytes can interact with GBM cells to improve their resistance to radiotherapy, but the impact on chemotherapy is not mentioned in the above study. Therefore, researchers have proposed a new hypothesis whereby astrocytes can transfer mitochondria through TNTs and improve chemotherapy resistance of GBM. Based on the results of previous studies, researchers have established 2D and 3D in vitro models simulating the tumor microenvironment,^60^ which can be used to study the impact of astrocytes on the GBM microenvironment. In the co‐culture system, thin‐film tubular structures were observed extending from UP‐010 astrocytes to UP‐007GBM cells. These structures contained F‐actin, and actin components unique to TNTs. Mitotracker orange was used to label mitochondria in astrocytes, and it was found that in both the 2D and 3D models, mitochondria can transmit through the above TNT structure.^61^ This mitochondrial transfer leads to an increase in the proliferation capacity of GBM cells and reduces the drug sensitivity of GBM to the chemotherapeutic drugs temozolomide (TMZ), vincristine (VCR), and clomipramine (CLM).

Glioma recurrence is dominated by glioma stem cells (GSCs) and similar to normal stem cell types, GSCs can also proliferate rapidly to achieve the ultimate goal of tumor growth.^62^ GSCs can also transfer mitochondria through TNTs, where studies used GBM stem cell‐like cells (GSLC) from the external infiltrating area of the GBM in one patient, and established 2D and 3D cell culture models. The results showed that the patient‐derived GSCs could form a TNT structure, which was positive for their characteristic F‐actin protein. Next, researchers stained the inner mitochondrial membrane with MitoGFP and determined that there was mitochondrial transfer in the TNT structure.^63^ Finally, they verified the effect of radiation on the formation of TNTs and mitochondrial transfer. Interestingly, different GSCs have different sensitivities to radiation. A part of the GSCs increases the formation of TNTs and mitochondrial transfer after being irradiated thus resisting radiation. Another part of the GSCs is sensitive to radiation, which may be caused by the coexistence of different molecular distributions and different therapeutic responses.^64^ Whatever the reason, TNT structure also exists in the GSCs and can transfer mitochondria. Radiation can cause different changes, thus further research is still needed to verify this.

### 3.2 Extracellular vesicles

Extracellular vesicles (EV) are a heterogeneous group of secretory membranous vesicles with a diameter of 30–1000 nm and are released from cells. They have different biogenesis, biophysical properties, and functions and can carry various biological molecules and participate in the physiological and pathophysiological processes of cells through intercellular communication.^65^ According to their morphological and biochemical characteristics, EVs can be divided into three subtypes, exosomes, microvesicles (MV), and apoptotic bodies.^66^ Exosomes are vesicles with a diameter in the range of 30–150 nm and are produced inside the cell, they then fuse with the plasma membrane and are released into the extracellular space.^67^ Microvesicles are vesicles that are shed by cell membranes and are released into the cytoplasm mediated by clathrin, typically they have diameters ranging from about 50–1000 nm. Apoptotic bodies are the products of programmed cell death, and their largest diameter is 5000 nm. EVs participate in biological processes such as cell proliferation, differentiation, and migration, and play an important role in the occurrence and development of cancer.^68^ There are many types of cargo that can be transported by EVs, including nucleic acids, proteins, and various metabolites, and mitochondria. Some studies have shown that donor cell‐derived outer vesicles containing mitochondria can fuse with macrophages transferring their mitochondria to the macrophages and changing their phagocytic function.^69^

NSCs mentioned earlier and can be used to treat degenerative diseases of the central nervous system, where these cells can transfer functional mitochondria by releasing EVs to restore the dysfunctional mitochondrial of the target cells. Proteomics has shown that mitochondrial proteins in EVs released by NSCs were significantly enriched and morphological and functional analysis confirmed that the EVs contained mitochondria with complete structure and function. After these EVs were fused by inflammatory mononuclear phagocytes, the expression of pro‐inflammatory markers in the target cells was reduced and normal mitochondrial dynamics was restored. Researchers constructed an animal model of multiple sclerosis and induced NSCs to transfer mitochondria to mononuclear phagocytes through microvesicles. The results showed that the clinical defects seen in the animals were significantly improved. It has also been shown that NSCs can transfer functional mitochondria to monocyte macrophages through EVs and this helped to reverse the mitochondrial dysfunction in nerve cells of patients with multiple sclerosis and other neurodegenerative diseases.^70^

### 3.3 Endocytosis

Endocytosis is a process in which eukaryotic cells form vesicles through the invagination of the cell membrane and take up extracellular substances into the cell, and constitutes two types phagocytosis and pinocytosis.^71^ In the glioma cell environment, mitochondria released by astrocytes can also be taken up by glioma cells through endocytosis. Mitochondria isolated from astrocytes labeled with mitotracker red was added to the induced starvation U87 glioblastoma cell line and In vivo fluorescence imaging showed that mitotracker red labeled mitochondria were present in the starved U87 cells. Researchers found that after starving U87 cells for 2 h, some extracellular substances could enter the cells through the invagination of the plasma membrane. Transmission microscopy confirmed that the plasma membrane of these inner lines contained mitochondria derived from astrocytes. After endocytosis of the mitochondria, the gene expression profile of U87 cells changed, whereby the genes related to glycolysis were up‐regulated, and the corresponding genes related to oxidative phosphorylation were down‐regulated. At the same time, the energy metabolism phenotype also changed. This is different from the results of a study we mentioned earlier, where the biological effect of mitochondria after entering U87 cells was to rescue the aerobic respiration of glioma cells, weaken the Warburg effect, and enhance the radiosensitivity of glioma cells.^72^ This contradictory finding may have been caused by different in vivo and in vitro environments. This experiment was carried out in vitro, and therefore cannot fully replicate the tumor microenvironment of the glioma patients. Therefore, there must be other factors affecting the energy metabolism of glioma cells in the tumor microenvironment and therefore the effect of mitochondrial metastasis on glioma cells needs further verification.

### 3.4 Gap junction

Gap junctions (GJ) are composed of gap junctional proteins (connexin, Cx), connexons, and gap junction channels (GJC), which are important for material exchange and signal exchange between adjacent cells.^73^ Six identical Cxs on the cell membrane surround a linker with a tubular structure. The two linkers on the adjacent cell membrane are connected end‐to‐end to form GJS. The GJC formed in the middle can allow ions, second messengers, sterols, phospholipids, and other small molecules to exchange across between cells. In vertebrate cells, Cx is a homologous transmembrane protein encoded by a multigene family. At present, more than 20 Cx subtypes have been found, of which Cx43 is the most widely expressed and thoroughly studied, where it participates in a series of physiological processes, such as material exchange, vesicle transport, mitochondrial respiration, and ion transport.^74^ Bone marrow mesenchymal stem cells (BMSC) protect injured neurons from apoptosis after spinal cord injury (SCI), which is achieved by transferring mitochondria through gap junctions. Researchers co‐cultured primary rat bone marrow mesenchymal stem cells with primary cortical neurons damaged by oxygen‐glucose deprivation (OGD). Using confocal microscopy, it was found that mitochondria from BMSCs stained with mitogen red could be transferred to damaged neurons. Immunoblot analysis proved that gap junction connexin 43 was expressed in BMSCs and that gap junction 32 was expressed in neurons, meaning that they may form heterotypic gap junctions through connexin 43 and connexin 32 between BMSCs and neurons and rely on this connection to transfer mitochondria. After accepting mitochondria, the biological spectrum of the neurons can be improved, and the survival rate can also be improved, with a downward trend in the expression of apoptosis‐related proteins.^75^ These experiments show that BMSCs protect neural cells from apoptosis by transferring mitochondria to damaged neurons through GJC (Figure 1C).

### 3.5 MitoCeption

The above‐mentioned methods of mitochondrial transfer between cells are all stimulated by certain factors. Researchers proposed a scheme of artificially quantitatively transferring mitochondria, termed MitoCeption. Here firstly, the mitochondria of MSC and GSCs were labeled and the GSCs were then subsequently cultured and the mitochondria in the MSCs were isolated. The Mitoception method was then used to transfer the isolated mitochondria into the GSCs, and FACS and confocal imaging were used to analyze metastasis. This mitochondrial transfer scheme applies to GSCs that grow as neurospheres in vitro. At the same time, cells carrying transferred mitochondria can be further analyzed to determine the impact of exogenous mitochondria on biological characteristics, such as cell metabolism, plasticity, proliferation, and response to treatment.^76^

## 4 MOLECULAR SIGNALING PATHWAYS INVOLVED IN INTERCELLULAR MITOCHONDRIAL TRANSFER IN THE CENTRAL NERVOUS SYSTEM

### 4.1 Miro1‐mediated mitochondrial transfer

Miro1 (mitochondrial Rho‐GTPase 1) is a calcium‐sensitive adaptor protein and its role is to connect mitochondria and kIF5 actin and assist mitochondria to move along microtubules in TNTs aided by a group of auxiliary proteins: Miro2, kinesin TRAK1, TRAK2, myosin Myo10, and Myo19.^77^ Miro1 has been found to play a key role in the axonal transport of neuronal mitochondria, and abnormalities in Miro1 exist in a variety of neurological diseases and mental illnesses.^78^ Miro1 is a major protein involved in the intercellular transport of mitochondria through TNTs; it does not affect the structural formation of TNTs but does affect the speed of transfer of mitochondria within the TNTs.^79^ The Ca^2+^ content in the mitochondrial matrix is related to the mitochondrial speed. Miro1 mainly affects speed by regulating the uptake of Ca^2+^ by mitochondria.^80^

Researchers have found that Miro1 levels increased in MMSC co‐cultured with neurons undergoing OGD. To explore whether Miro1 levels are an important factor affecting mitochondrial intercellular transport, researchers constructed a lentiviral vector containing the Miro1 gene and introduced it into MMSCs, resulting in increased Miro1 expression. When these Miro1‐MMSCs were co‐cultured with astrocytes, the latter received increased mitochondria from the MMSCs.^22^ It was also found in an in vitro model simulating cerebral ischemia that MMSCs overexpressing Miro1 showed stronger mitochondrial transfer ability and a greater ability to repair cell damage. However, after transfecting astrocytes with siRNA to knock down Miro1, the astrocytes reduced the number of mitochondria transferred to cisplatin‐treated neurons and prevented the normalization of neuronal calcium dynamics.^28^

### 4.2 cADPR/Ca^2+^‐Mediated Mitochondrial Transfer

Cyclic ADP‐ribose (cADPR) is a second messenger that can activate the endoplasmic reticulum and mobilize the release of intracellular Ca^2+^, an important signal involved in triggering cell endocytosis and exocytosis. CD38, a member of the NAD^+^ glycolytic enzyme family, catalyzes the synthesis of calcium messenger loop ADP ribose (cADPR) in the mitochondrial membrane.^81^ In the nervous system, CD38 is expressed in glial cells to regulate their metabolic processes^82^ and rat cortical astrocytes can express CD38 protein and have cADPR cyclase activity. When a Crispr/Cas9 activation plasmid was used to upregulate CD38 in astrocytes, the number of extracellular mitochondria in the conditioned medium increased. In a mouse model of simulated cerebral ischemia, researchers detected CD38 upregulated in the cortex.^27^ Therefore, it can be concluded that neuronal ischemic injury caused by adverse events stimulates peripheral astrocytes to upregulate the level of intracellular CD38 protein, which catalyzes the transformation of more NAD^+^ to cADPR, thereby increasing the level of Ca^2+^ in astrocytes, promote the movement of mitochondria to the cell membrane, and promote the formation and release of extracellular vesicles. These vesicles carry functional mitochondria, close to damaged neurons, and transfer them to the neurons by endocytosis, promoting the recovery of ischemic neuronal activity and reducing neuronal death (Figure 1A). CD157 (BST‐1, bone marrow stromal antigen‐1) is a cell surface molecule expressed in endothelial mesothelial cells and other cells. Like CD38, it also belongs to the NADase/ADP ribocyclase family^83^ and can act as a signal receptor for signal transduction. Previous studies have shown that CD157 can promote the expansion of pro‐T cells and participate in cellular and humoral immune responses.^84^ Since it can also catalyze the conversion of NAD^+^ to cADPR, it can also participate in the regulation of mitochondrial transport. When BMSCs were co‐cultured with injured VSC4.1 motor neurons, the expression of CD157 in the BMSCs increased. Then the BMSCs were transfected with CD157 overexpressing vector and a CD157 interference vector, and the results showed that the BMSCs overexpressing CD157 could release more mitochondria to the outside of the cell. Similarly, BMSCs with low CD157 expression had a significantly reduced extracellular mitochondrial content. The upregulation of CD157 in BMSCs can also promote the transfer of extracellular mitochondrial particles to VSC4.1 motor neurons, gradually regenerating the VSC4.1 motor neuron axons and reducing apoptosis. To determine whether CD157 regulates mitochondrial release through the cADPR/Ca^2+^ pathway, researchers evaluated the activity of cADPR and the intracellular calcium level in BMSC cells. The results showed that the highest cADPR activity and the highest intracellular Ca^2+^ concentration were found in BMSCs with high expression of CD157.^85^ Therefore, CD157, like CD38, participates in intercellular mitochondrial transport through the CD157‐cADPR‐Ca^2+^ signaling pathway.

Both the above‐mentioned pathways are examples of modulation of mitochondrial donor cells, and the CD38‐cADPR‐Ca^2+^ pathway can also affect the endocytosis of mitochondria containing vesicles by recipient cells. FK‐506 binding protein 12.6 (FKBP12.6) can bind to ryanodine receptors (RYR) found on the endoplasmic reticulum (ER) and inhibit the activity of the RYR (intracellular calcium release channel).^86^ cADPR can induce FKBP12.6 to dissociate from the RYR, leading to the release of Ca^2+^ from the calcium stored within the endoplasmic reticulum. High levels of binding of cytosolic Ca^2+^ to troponin C triggers conformational changes in F‐actin, leading to cytoskeletal remodeling, promoting plasma membrane invagination, and causing endocytosis.^87^ Studies have also found that astrocytes can transfer mitochondria to OGD glioblastoma U87 cells. In these cells, the conversion of NAD^+^ to NADH decreased, and a large amount of accumulated NAD^+^ was released into the extracellular space. CD38 catalyzes the cyclization of extracellular NAD^+^ to intracellular cADPR. This triggers the release of calcium ions, promoting cytoskeleton remodeling and plasma membrane invagination, thus increasing the rate of endocytosis of mitochondrial vesicles in U87 cells, and weakening the Warburg effect of U87 cells.

### 4.3 SCL1A5/MFSD2 and HERV‐WE1/HERV‐FDR1

Human endogenous retrovirus (HERV) exists in the human genome, accounting for about 8% of genetic material.^88^ HERV elements have the characteristics of retroviruses, including the presence of non‐coding long‐terminal repeat genes on both sides. HERV can be divided into three categories according to the sequence homology of external viruses: class I, broadly clustering with ε (epsilon) and γ (gamma) viruses, Class II, clustering with β (beta) viruses, and Class III, the members of which are most closely related to spumaviruses.^89^ Although most HERVs lose the ability of horizontal expression transmission due to mutations and epigenetic modifications, there remain some genes that can encode corresponding proteins when cells are in a pathological state,^90^ and these proteins are considered to be involved in cell transformation and cancer development. The enhanced expression of specific HERV proteins has been found in different tumors, some of which seem to contribute to the development of cancer, and some mechanisms of action have been proposed.^91^ HERV envelope proteins WE1 (syncytin‐1) and FRD1 (syncytin‐2) seem to be highly related to mitochondria and may even promote their intercellular exchange through free uptake across cell membranes. When U87 glioblastoma cells were treated with the cytotoxic drug etoposide, it was observed that mitochondria were clustered around the nucleus, while herv‐frd1 and herv‐we1 were also localized in the perinuclear space. Western blot was used to detect the mitochondrial protein extracts isolated from glioblastoma and it was found that the mitochondrial membrane contained herv‐we1 and herv‐frd1. In addition, the corresponding receptors, ASCT2 and MFSD2, were also expressed on the mitochondrial membrane. On the membrane of U87 glioblastoma cells acting as receptors, HERV‐FRD1, HRRV‐WE1, and their corresponding receptors ASCT2 and MFSD2 were also expressed. When the extracellular mitochondria migrate to U87 cells, HERV acts as a ligand and binds to the corresponding receptors on the U87 cell membrane to promote the direct transmembrane uptake and purification of mitochondria from donor cells. Anti‐syncytin‐1 and anti‐syncytin‐2 antibodies are antibodies that can target homologous receptors to specifically block the direct uptake of mitochondria by cells. In chemotherapy refractory cancer cells, this may open an attractive avenue for new mitochondrial‐targeted therapies.^92^

## 5 TARGETS AND CORRESPONDING DRUGS FOR INTERFERENCE WITH INTERCELLULAR MITOCHONDRIAL TRANSFER IN THE CENTRAL NERVOUS SYSTEM

### 5.1 TNT inhibitors

TNTs have been shown to be the main method by which MSCs can transfer mitochondria or other organelles to other cells.^93^ TNTs are composed of cell membranes and their cytoskeleton is composed of F‐actin and microtubulin. Therefore, F‐actin polymerization plays an important role in the structural formation of TNTs.^94^ LatA, an inhibitor of F‐actin polymerization, was used in experiments to demonstrate the formation and function of TNTs. Annexin V can protect phosphatidylserine exposed on the surface of damaged endothelial cells and inhibit the formation of intercellular TNTs. In one study, researchers showed that LatA or annexin V can significantly reduce the number of rescued host cells in and around the microvessels from a periinfarcted area.^95^ When MSCs were co‐cultured with cerebral vascular endothelial cells simulating ischemia, it was found that the MSCs transferred mitochondria to endothelial cells through TNTs. However, after the addition of LatA or annexin V, the formation of TNTs was significantly reduced, resulting in the failure of damaged cells to recover their normal respiratory and metabolic activities.^96^ This TNT inhibitor, however, is not conducive to the recovery of disease for neurological dysfunction caused by ischemic injury of the nervous system or mitochondrial dysfunction. However, for glioma, inhibiting the formation of TNTs and then inhibiting the metastasis of mitochondria can prevent the Weinberg effect in these cells, which is not conducive to their proliferation. Therefore, TNT inhibitors may represent a candidate drug to target the treatment of brain malignant tumors in the future (Table 1).

**TABLE 1:** Drugs targeting the regulation of intercellular mitochondrial transport.

Drug name	Pharmaceutical structure	Target location	Pharmaceutical affection	Affected method
Latrunculin A	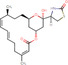	F‐Actin	Destroy actin polymerization, prevent mitotic spindle formation, and affect cytoskeleton	TNT
Annexin V	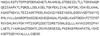	F‐Actin	Protect phosphatidylserine exposed on the surface of damaged endothelial cells and inhibit the formation of intercellular TNT	TNT
SB203580	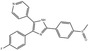	p38	Inhibitors of p‐38 which can reduce the level of p‐p38 in cells, inhibition of vesicle formation and release through the MAPK pathway	Vesicle
Retinoic Acid	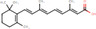	Cx43	Stimulate the physical binding of PP2A and Cx43, change the phosphorylation state of Cx43 protein, reduce phosphorylated Cx43, increase nonphosphorylated Cx43, and upregulate gap junction level	GJC
18 β Glycyrrhetinic Acid	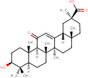	Cx43	Induce phosphorylation of Cx43, reduce the level of nonphosphorylated Cx43, and inhibit gap junction between cells	GJC

### 5.2 Vesicle inhibitors

Parkinson's disease (PD) is a progressive neurological disease that affects movement, with symptoms including resting tremor, bradykinesia, rigidity, and non‐motor symptoms. Parkinson's disease is characterized by the progressive loss of dopaminergic (DA) neurons in the substantia nigra (SN) region of the brain and the presence of Lewy bodies (LB).^97^ In an in vitro Parkinson's disease model, it was found that astrocytes derived from human umbilical cord‐derived stem cells could transfer mitochondria to damaged neurons in PD, and this transfer pathway was performed by extracellular vesicles. This p38‐MAP kinase‐regulated endocytosis is mediated by clathrin or Rab5, where the complex is dependent upon endocytic trafficking. The researchers used rotenone to treat dopamine neurons to simulate the neuron‐like state seen in patients with Parkinson's disease. Western blot analysis showed that the level of phosphorylated p38 (pp38) was significantly increased in rotenone‐treated DA neurons. SB203580 is an inhibitor of P‐38, which can reduce its intracellular concentration^98^ and when this was used, the number of mitochondria transferred from astrocytes to DA neurons was significantly reduced, and even the rotenone‐damaged DA neurons could not be rescued.^99^ Therefore, p38/MAPK pathway inhibitors can be regarded as a class of specific targeted drugs for the pathway associated with the vesicular transport of mitochondria (Table 1).

### 5.3 Gap junction activators and inhibitors

BMSCs can transfer mitochondria into damaged motor neurons and there are many ways for this transfer, including TNT (the most common type), but also intercellular gap junctions. The transferred mitochondria can reduce the apoptosis of motoneurons and promote their functional recovery. Connexin proteins are an important structural component of gap junctions and Cx43 is one of the most important gap junction protein family members involved in material exchange. Live cell imaging studies have found that GJC composed of Cx43 is formed between bone marrow‐derived MSCs and injured alveolar epithelial cells, and the vesicles that encapsulate mitochondria released by MSC reach alveolar epithelial cells through GJCs and then are taken up by endocytosis.^100^ The transfer of mitochondria to lung epithelial cells through GJCs is one of the important mechanisms of MSC for treating lung injury. Retinoic acid (RA) can change the phosphorylation state of Cx43 protein, so that phosphorylated Cx43 decreases and non‐phosphorylated forms increase. This process is dependent on protein phosphatase 2A (PP2A) activity. RA can stimulate the physical binding of PP2A to Cx43, leading to the dephosphorylation of Cx43, thereby upregulating gap junction abundance.^101^ 18 β Glycyrrhetinic acid (18 β GA), a saponin isolated from the root of Glycyrrhiza uralensis, has been shown to be an inhibitor of gap junction communication. 18βGA can induce phosphorylation of Cx43, which plays an opposite role of RA and inhibits gap junctions between cells.^102^ These experimental results show that 18 βGA inhibits GJC, thereby reducing mitochondrial transfer from BMSC to damaged motor neurons, while RA activates GJIC, thereby increasing mitochondrial transfer (Table 1).

### 5.4 Exogenous transplantation

Mitochondrial transport plays an important role in maintaining cell homeostasis and cell function. There is growing evidence that many degenerative diseases, such as lung disease, cardiomyopathy, and brain injury, are strongly associated with mitochondrial dysfunction.^103^ Therefore, it becomes extremely urgent to explore how to positively adjust the function of mitochondria. It is widely recognized potential strategy is to artificially replace damaged mitochondria in diseased cells with healthy mitochondria to maintain mitochondrial homeostasis.^100^ However, this strategy faces challenges in maintaining mitochondrial integrity.^104^ Although it has not been proven in central nervous system diseases, it has been demonstrated in lung degenerative diseases that using iron oxide nanoparticles (IONPs) can selectively enhance the transfer of intercellular mitochondria from human mesenchymal stem cells (hMSCs) to diseased cells in order to restore the potential of mitochondrial bioenergy.^105^ This might be a potential way to improve the efficiency of mitochondrial transfer.

## 6 CONCLUSIONS AND FUTURE EXPECTATIONS

Cells under stress or injury can emit various distress signals, including chemical signals such as cytokines and abnormal metabolites, and electrical signals such as changes in membrane potential. After stimulation by these distress signals, other cells in the surrounding environment adjacent to these cells can be recruited to the damaged cells and establish complex intercellular communication with them for effective material and information exchange. The most important and common example is the intercellular transfer of mitochondria. The effect of establishing communication is to affect metabolic levels and energy programming in the damaged or stressed cells. For different types of cell damage caused by different stimuli, this metabolic reprogramming is beneficial to the cells. However, for the whole human disease state, some types of cell communication are often one of the reasons for the poor prognosis of patients. In terms of neurological ischemic diseases or neurodegenerative diseases caused by ischemia and hypoxia,^34^ the transfer of mitochondria from healthy cells to damaged cells can often restore their activity, rescue mitochondrial respiratory function, promote oxidative phosphorylation, reduce lactic acid production, and finally avoid apoptosis. For nervous system tumors, mitochondrial transfer seems to be a detrimental phenomenon. Under the influence of the Weinberg effect, tumor cells seem to prefer to maintain the high energy demand of cells through aerobic glycolysis.^106^ Therefore, the transfer of mitochondria promotes tumor cells to run aerobic glycolysis to a greater extent, thus promoting their proliferation and enhancing sensitivity to radiation and chemical drugs, which is not conducive to the follow‐up treatment of patients. At present, the most common cell type used as a mitochondrial donor is MSCs.^107^ Separating active mitochondria from MSCs and directly injecting them into damaged lesions may represent a novel future disease treatment. Purification of mitochondria from murine‐derived stem cells and their injection into human neural cells can change the metabolic state of neural cells.^108^ Therefore, in the face of different types of nervous system diseases, the correct use of mitochondrial transfer enhancers or inhibitors and the protection of mitochondrial activity are urgent problems to be resolved in clinical trials. In the future, the combined application of multiple mitochondrial transfers targeting drugs may improve the prognosis of various central nervous system diseases.

## AUTHOR CONTRIBUTIONS

Conception and design: Geng ZA, Zhu C. Acquisition of references: Geng ZA, Guan S, Wang SQ, Yu ZX. Analysis and interpretation of data: Geng ZA, Guan S, Wang SQ. Drafting the article: Geng ZA, Guan S, Wang SQ, Yu ZX. Critically revising the article: Zhu C, Du SN, Liu TC. Reviewed submitted version of manuscript: Zhu C, Du SN, Liu TC. Approved the final version of the manuscript on behalf of all authors: Zhu C. Study supervision: Zhu C.

## FUNDING INFORMATION

This study was supported by the National Natural Science Foundation of China (no. 82103450 to Zhu C).

## CONFLICT OF INTEREST STATEMENT

The authors have no personal, financial, or institutional interest in any of the drugs, materials, or devices described in this article.

## CONSENT FOR PUBLICATION

All authors gave their consent for publication.

## Data Availability

Data sharing is not applicable to this article as no new data were created or analyzed in this study.
